# CXCR4 Antagonism to Treat Delayed Fracture Healing

**DOI:** 10.1089/ten.tea.2018.0265

**Published:** 2019-09-17

**Authors:** Richard Meeson, Anita Sanghani-Keri, Melanie Coathup, Gordon Blunn

**Affiliations:** ^1^ Division of Surgery, Institute of Orthopaedics and Musculoskeletal Science, University College London, London, United Kingdom.; ^2^ Department of Clinical Services and Sciences, Royal Veterinary College, Hatfield, United Kingdom.; ^3^ University of Central Florida, Orlando, Florida.; ^4^ University of Portsmouth, Portsmouth, United Kingdom.

**Keywords:** delayed union, endogenous mobilization, AMD3100, CXCR4, fracture healing, external fixator

## Abstract

A significant number of fractures develop nonunion. Stem cell homing is regulated through stromal cell-derived factor 1 (SDF1) and its receptor CXCR4. Stem/progenitor cell populations can be endogenously mobilized by administering growth factors with a pharmacological antagonist of CXCR4, AMD3100, which may be a means to improve fracture healing. A 1.5 mm femoral osteotomy in Wistar rats was stabilized with an external fixator. Rats were pretreated with phosphate buffered saline [PBS(P)], vascular endothelial growth factor [VEGF(V)], insulin-like growth factor-1 [IGF1(I)], or granulocyte colony stimulating factor [GCSF(G)] before AMD3100. A control group (C) did not receive growth factors or AMD3100. Bone formation after 5 weeks was analyzed. Group P had a significant increase in total bone volume (BV) (*p* = 0.01) and group I in percentage bone in the fracture gap (*p* = 0.035). Group G showed a decrease in BV. All treated groups had an increase in trabecular thickness. Histology showed decreased cartilage tissue associated with increased bone in groups with improved healing, and increased fibrous tissue in poorly performing groups. Antagonism of SDF1-CXCR4 axis can boost impaired fracture healing. AMD3100 given alone was the most effective means to boost healing, whereas pretreatment with GCSF reduced healing. AMD3100 is likely mobilizing stem cells into the blood stream that home to the fracture site enhancing healing.

**Impact Statement:**

Currently ∼10% of fractures progress to delayed or nonunion with significant morbidity and economic impact. Endogenous mobilization of stem cells by pharmacological antagonism of their homing and migration receptor CXCR4 with AMD3100 experimentally reduced delayed union development. Endogenous mobilization may, therefore, translate as a low risk means to boost healing and could potentially be given as a prophylaxis to patients with fractures at risk of delayed healing or nonunion. These patients may include fragility fractures, comminuted tibial fractures, or when treating established nonunions. This approach could have promise for other conditions that may benefit from stem cell treatments.

## Introduction

Asignificant number of bone defects and fractures do not heal,^1^ with estimates of ∼100,000 fractures per year developing nonunion in the United States.^2^ To achieve bone union, there is a need to recruit a range of cells, including inflammatory cells, endothelial cells, and stem cells, from a range of tissue sources, including muscle, bone marrow, adipose tissue, and periosteum.^3^ At rest there are low basal levels of peripherally circulating skeletal progenitors; ∼1 per 10^6–8^ blood mononuclear cells,^4–6^ whereas the numbers of levels of mesenchymal stem cells (MSCs)^7^ and endothelial progenitor cells (EPCs)^8^ in the blood stream increase postbone fracture. The chemokine stromal cell-derived factor 1 (SDF1; also known as CXCL12) and its receptor CXCR4 have a key role in stem cell migration from the bone marrow stroma into the circulation and are believed to be important for homing of stem cells to a fracture site.^9^ Local increases in SDF1 expression have been measured in distraction osteogenesis, stress fractures, and segmental defects.^10–12^ Parabiotic studies have demonstrated that labeled stem cell mobilization is from the bone marrow through the peripheral circulation and these cells are able to then contribute to the fracture healing process.^13,14^

Mobilization of hematopoietic stem cells is a mainstay of clinical bone marrow transplantation to treat a range of blood-related malignancies, and granulocyte colony stimulating factor (GCSF) was the first growth factor used for this purpose.^15^ A highly selective high-affinity competitive antagonist of the CXCR4 receptor, AMD3100,^16^ commercially known as Mozibil™, rapidly mobilizes high numbers of hematopoietic stem cells by blocking their interaction with SDF1 in the marrow niche.^17–19^ It has been shown in mice that when AMD3100 is given after pretreatment with vascular endothelial growth factor (VEGF) rather than GCSF, there is preferential mobilization of a population of MSCs and EPCs relative to hematopoietic stem cells.^20^

To date, a few groups have started to investigate mobilization of stem cells to augment bone healing. Critical-sized calvarial defects have shown enhanced healing with 15 daily injections of AMD3100 in mice,^21^ and a single dose of AMD3100 was also shown to improve intramedullary trabecular bone reformation.^22^ For evaluation of diaphyseal long bone healing, Kumar and Ponnazhagan^23^ mobilized MSCs by pretreatment with insulin-like growth factor-1 (IGF1) followed by AMD3100, in a coapted mouse tibial segmental fracture, and showed a significant increase in bone mineral density. AMD3100 has also been evaluated after creating an “Einhorn style” mouse femoral fracture stabilized with a single intramedullary pin, and fracture healing was accelerated.^24^

None of the aforementioned studies allow for direct comparison of the different protocols, and neither do they test the effect of endogenous mobilization in a translationally relevant delayed union model. Therefore, based on current literature, this study aimed to compare different mobilization protocols (AMD3100 alone, or after pretreatment with GCSF, IGF1, or VEGF) in a biomechanically controlled, delayed union fracture model, of a rat femoral osteotomy stabilized using an external fixator.^25^ The hypothesis was that antagonism of the SDF1/CXCR4 axis using ADM3100 would improve bone healing in a delayed union fracture model, and pretreatment with IGF1 or VEGF before AMD3100 would have the greatest efficacy due to their preferential MSC mobilization.

## Methodology

### Growth factor and AMD3100 preparation

AMD3100 octahydrochloride hydrate (A5602; Sigma-Aldrich, UK) stock solution was prepared by dissolving 5 mg lyophilized product in 0.5 mL sterile water, then added to 4.5 mL phosphate buffered saline (PBS) to produce a 1 mg/mL injection solution, which was aliquoted and stored at −20°C until needed. Rat VEGF 165 (400-31; PeproTech, Rocky Hill, NJ) was prepared by dissolving the lyophilized product in sterile water to make a 0.1 mg/mL stock solution and then 1 mL of stock solution was added to 4 mL of sterile PBS +0.1% bovine serum albumin (BSA) (A9418; Sigma-Aldrich) to achieve 100 μg/mL injectable solution, which was aliquoted and stored at −20°C until needed. Recombinant human IGF1 (100-11; PeproTech) and murine GCSF (250-05; PeproTech) were prepared in the same manner. Finally, PBS +0.1% BSA, “sham growth factor,” to determine the effects of AMD3100 alone was also prepared.

### Fracture model

A total of 12- to 14-week-old female Wistar rats (230–300 g) were randomly assigned to one of the five groups: PBS+AMD3100 (*n* = 6; PBS-AMD), VEGF+AMD3100 (*n* = 8; VEGF-AMD), IGF1+AMD3100 (*n* = 6; IGF1-AMD), GCSF+AMD3100 (*n* = 6; GCSF-AMD), and non-mobilized control fracture group (*n* = 7). A linear type 1a micro-external fixator, with titanium blocks and carbon fiber bars was placed on the left craniolateral femur after a lateral surgical approach.^25^ Using a precision guide, four bicortical 1.4 mm diameter end-threaded self-tapping stainless steel pins were placed in predrilled 1.0 mm holes. Consistent proximodistal positioning was based on the distal extent of the greater trochanter. Pins were exited through separate skin incisions and the custom variable spacing fixator was attached.^25^

A mid-diaphyseal femoral osteotomy with no periosteal stripping was made using a diamond tipped handsaw, while applying sterile saline coolant/lubricant. A precision spacer ensured a fixed distance between the cis cortex and connecting blocks of 9 mm. The fixator was then used to distract the osteotomy gap to 1.5 mm using a second precision spacer. The biceps femoris was closed over the osteotomy with a single horizontal mattress suture (1.5M PDS II; Ethicon, UK), and then the skin was closed with intradermal continuous suture (1.5M Monocryl; Ethicon). Activity was unrestricted postsurgery. In two rats the surgical wounds failed to heal and were removed from the study, leaving *n* = 5 for both the GCSF-AMD and PBS-AMD groups.

Twenty-four hours postsurgery, rats were given a single intraperitoneal (i.p.) injection of either VEGF, IGF1, GCSF, or PBS once daily for 4 days at 100 μg/kg.^20^ On day 5, they were given a single injection of AMD3100 at 5 mg/kg.^20–24^ All i.p. injections, including AMD3100 and sham PBS, were administered at a volume of 0.5 mL/100 g bodyweight based on the day 0 presurgical weight. Rats were sacrificed at 5 weeks postoperatively. All procedures were carried out with the local Animal Welfare and Ethical Review Body approval under personal and project licenses issues by the UK Home Office under the 1986 Animal Scientific Procedures Act.

### Microcomputed tomography and radiographic analysis

The left femur with the fixator in place was retrieved. To reduce microcomputed tomography (microCT) beam-hardening artifact generated from the interaction of the X-ray beam and the metallic implant, a radiolucent PEEK fixator block was connected externally to the fixator pins after careful removal of the skin with surrounding soft tissues, and then without disturbing the fracture callus the titanium block fixator was then removed. Samples were fixed in 10% buffered formaldehyde for up to 3 days. The formalin-fixed samples were wrapped in cling film to prevent dehydration and mounted into a sample holder for microCT scanning. Samples were scanned using a Bruker Skyscan 1172 microtomograph machine (Bruker, Belgium), at 60 kV, 167 μA with a 0.5 mm aluminum filter. A rotation step of 0.5°, without frame averaging, and an image pixel size of 4.89 μm was used. A single image capture image was taken with the image intensification “scout” before scanning, for two-dimensional (2D) radiographic assessment of the osteotomy union. Radiographic scouts were randomized and blinded to score the general impression of healing according to the AO-ASIF recommendations for long bone fractures; united, not united, or uncertain^26^ as follows: *ununited* where there was no mineralized tissue bridging between the ends of the osteotomy; *uncertain* where there was new bone formation; however, a radiolucent line remained between the proximal and distal segments, and *not united* where no gap between bone ends was visible.

MicroCT scans were reconstructed using NRecon (Bruker) with smoothing = 2, ring artifact reduction = 12% and beam hardening artifact = 41%. Analysis was performed with CTAn (Bruker). Using the measuring tool, the center point of the osteotomy was determined and the transverse slice at that point was selected as the reference slice. The central 60% of the osteotomy gap, that is, only new bone formation within the osteotomy was analyzed. The callus was isolated using a 2D region of interest shrink wrap stretching over holes <40 pixels, despeckled <150 voxels, and then three-dimensional (3D) analysis was performed.

### Histology preparation

Bones were decalcified in a 12.5% solution of ethylenediaminetetraacetic acid and sequentially dehydrated for 24 h, followed by defatting with chloroform for 48 h and then embedded into wax, with the fixator pins orthogonal to the facing surface of the block. Fixator blocks and pins were removed once the wax had set and a microtome (ThermoFisher Scientific, UK) was used to make 5 μm thick slices. The alignment of the blocks within the microtome was altered as necessary to ensure a central sagittal slice through the femur. The position of a mid-sagittal section through the fracture gap was assessed using the fixator pin tract holes. Wax slices were mounted onto positively charged glass slides (X-tra; Leica Biosystems, UK), dewaxed, and then hydrated. Samples were then stained with hematoxylin (Sigma-Aldrich) nuclear stain for 5 min. Excess stain was removed by gently washing with water for 5 min. Slides were counterstained in 1% eosin (Sigma-Aldrich) for 4 min and then washed and dehydrated in increasing concentrations of alcohol. Slides were cleaned in xylene and mounted under 40 mm coverslips using Pertex Mounting Medium (CellPath plc, UK).

### Histomorphometric analysis

Slides were observed under a light microscope (KS-300 Zeiss, UK). Histomorphometric analysis using the 2.5 × objective was performed on the most central slice, using a line-intercept method with a grid scaled to the graticule and drawn using PowerPoint (Microsoft, USA). The grid covered the entire visual field from top to bottom (lateral to medial cortex) and was centered over the osteotomy; its width was equivalent to the original 1.5 mm osteotomy. Grid “density” was 120 intersections and grid squares were 160 μm in both directions. Intersections were then scored as bone, cartilage, fibrous tissue, vascular (red blood cells seen not within tissue matrix), or void.

### Statistical analysis

As the data were nonparametric, analyses included the Mann–Whitney *U* and Kruskal–Wallis as appropriate. Significance was set at *p* < 0.05 and tests were analyzed with SPSS version 24 (IBM, Chicago, IL).

## Results

### Influence of mobilization on fracture healing

**FIG. 1.**
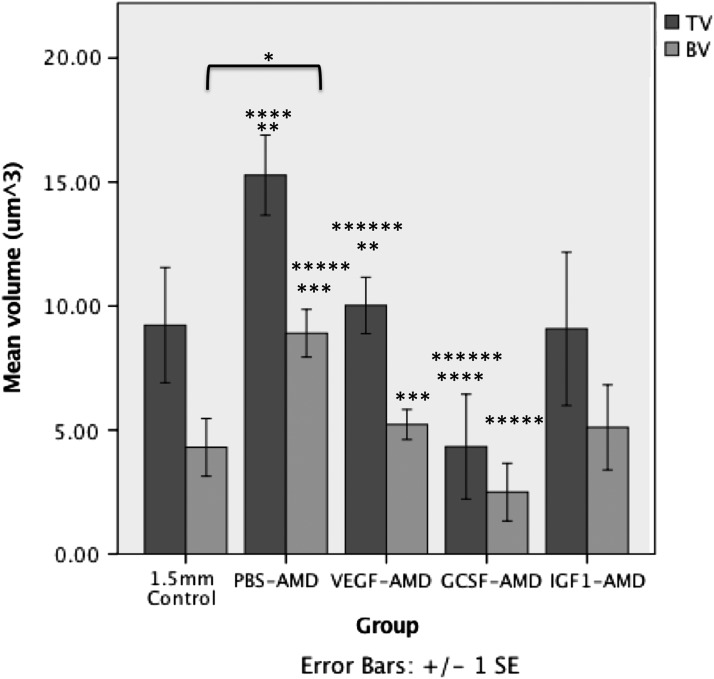
The mean ± SEM TV and BV within the osteotomy measured using microCT. *Represents significant (*p* < 0.05) differences compared with 1.5 mm control. **, ***, ****, *****, and ****** indicate significant differences (*p* < 0.05) between paired groups. BV, bone volume; GCSF, granulocyte colony stimulating factor; IGF1, insulin-like growth factor-1; microCT, microcomputed tomography; PBS, phosphate buffered saline; SEM, standard error of the mean; TV, tissue volume; VEGF, vascular endothelial growth factor.

Radiographic score showed a reduction in not united (nonunion) and increases in uncertain and united (union) for all groups compared with the GCSF-AMD group. Radiographically united fractures occurred in 4/5 animals in the PBS-AMD group, whereas this was reduced for the VEGF, IGF, and GCSF pretreated groups with control animals only showing a united rate in two animals out of seven (Table 1). MicroCT analysis showed that all groups other than GCSF-AMD had improved healing over the controls (Table 2). PBS-AMD had twice the bone volume (BV) within the osteotomy (8.9 ± 2.2 μm^3^, *p* = 0.01), compared with the untreated control (4.3 ± 3.1 μm^3^). Not only was the BV increased, but the overall callus tissue volume (TV) was increased compared with controls (15.3 ± 3.6 vs. 9.2 ± 6.1 μm^3^) (Table 2; Fig. 1).

**Table 1.** AO-ASIF Global Radiographic Healing Score from the Mediolateral Radiograph at 5 Weeks Postsurgery

*Group*	*Not united*	*Uncertain*	*United*
1.5 mm Control	3/7 (43%)	2/7 (29%)	2/7 (29%)
PBS-AMD	1/5 (20%)	0/5 (0%)	4/5 (80%)
VEGF-AMD	2/8 (25%)	2/8 (25%)	4/8 (50%)
GCSF-AMD	3/5 (60%)	0/0 (0%)	2/5 (40%)
IGF1-AMD	2/6 (33%)	1/6 (17%)	3/6 (50%)

GCSF, granulocyte colony stimulating factor; IGF1, insulin-like growth factor-1; PBS, phosphate buffered saline; VEGF, vascular endothelial growth factor.

**Table 2.** Microcomputed Tomography Quantitative Morphology Data from the Central 60% of the Osteotomy at 5 Weeks

	*1.5 mm Control*	*PBS-AMD*	*VEGF-AMD*	*GCSF-AMD*	*IGF1-AMD*
TV (μm^3^)	9.23 ± 6.14	15.28 ± 3.61	10.03 ± 3.22	4.33 ± 4.72	9.08 ± 7.57
BV (μm^3^)	4.31 ± 3.08	**8.91 ± 2.16**	5.22 ± 1.71	2.50 ± 2.60	5.11 ± 4.21
TV/BV (%)	53.79 ± 20.82	58.51 ± 6.06	52.52 ± 5.85	**63.07 ± 7.29**	**59.24 ± 5.58**
TS (μm^2^)	62.83 ± 45.55	60.32 ± 14.75	63.56 ± 19.88	34.24 ± 27.19	39.77 ± 30.77
BS (μm^2^)	326.15 ± 220.05	450.92 ± 121.44	355.52 ± 130.15	133.83 ± 147.25	269.57 ± 232.90
Tb.Th (μm)	0.04 ± 0.01	**0.06 ± 0.00**	0.05 ± 0.01	**0.07 ± 0.03**	**0.06 ± 0.01**
Tb.Sp (μm)	0.07 ± 0.03	0.09 ± 0.03	0.08 ± 0.02	0.06 ± 0.02	0.08 ± 0.03
Tb.N (1/μm)	14.09 ± 9.32	9.57 ± 1.01	10.99 ± 1.08	10.49 ± 4.88	9.71 ± 1.30
TotPor (%)	46.21 ± 20.82	41.49 ± 6.06	47.48 ± 5.85	**36.93 ± 7.29**	**40.76 ± 5.58**

Significant results (p < 0.05) are shown in bold.

BS, bone surface area; BV, bone volume; BV/TV, percentage bone volume; TotPor, total porosity; Tb.Sp, trabecular separation; Tb.Th, trabecular thickness; TS, tissue surface area; TV, tissue volume.

**FIG. 2.**
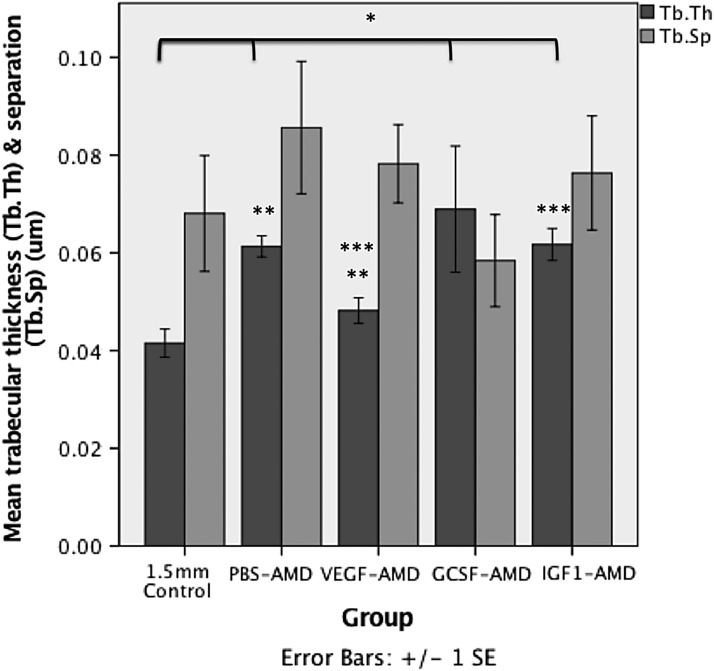
The mean ± SEM Tb.Th and Tb.Sp distance of bone formed within the osteotomy measured using microCT. *Represents significant (*p* < 0.05) differences compared with 1.5 mm control. ** and *** indicate significant differences (*p* < 0.05) between different groups. Tb.Sp, trabecular separation; Tb.Th, trabecular thickness.

The percentage bone volume (BV/TV) was not significantly increased owing to a relative proportional increase in bone and nonmineralized callus tissue. In addition, the bone structure was different in the PBS-AMD group compared with controls. Animals in the PBS-AMD group showed a significant increase in trabecular thickness (Tb.Th), 0.061 ± 0.002 μm compared with those in the control group, which had a thickness of 0.042 ± 0.003 μm (*p* = 0.03) (Table 2; Fig. 2).

The VEGF-AMD group did not show any significant differences from the control group. Interestingly, animals in the GCSF-AMD group had a significant increase in BV/TV 63.1 ± 7.3% versus 53.8 ± 20.8% (*p* = 0.048), but the actual TV (4.3 ± 4.7 vs. 9.2 ± 6.1 μm^3^) and BV (2.5 ± 2.6 vs. 4.3 ± 3.1 μm^3^) was reduced compared with controls. However, Tb.Th was significantly higher 0.069 ± 0.03 versus 0.042 ± 0.008 μm (*p* = 0.048) (Fig. 2), but total porosity (TotPor) was significantly lower 36.9 ± 7.3% versus 46.2 ± 20.8% (*p* = 0.048), indicating that although GCSF-AMD group had less overall total woven bone, the bone formed was less porous and the size of each bone forming region within the fracture gap was larger than in controls.

IGF1-AMD group also had an increase in BV (5.1 ± 4.2 μm^3^) compared with controls. BV/TV was significantly increased (*p* = 0.035) and the overall callus size was the same as controls (TV 9.1 ± 7.6 vs. controls 9.2 ± 6.1 μm^3^). There was also a significant increase in Tb.Th 0.062 ± 0.008 μm (*p* = 0.01) (Fig. 2). TotPor was significantly lower within the fracture gap of animals treated with IGF1-AMD, compared with controls; 40.8 ± 5.6% versus 46.2 ± 20.8% (*p* = 0.035). The spread of data was not significantly different.

**FIG. 3.**
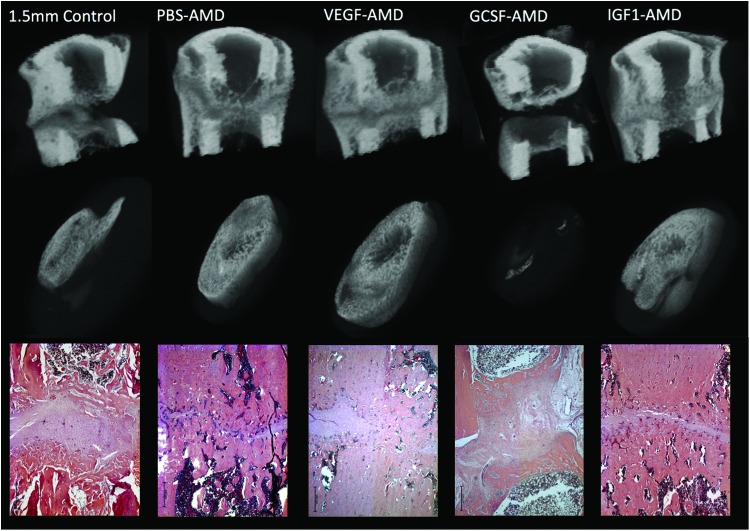
MicroCT 3D reconstructions of mid-femoral regions, with a mid-sagittal reveal (*top row*). The *middle row* shows a 3D reconstruction of the central 60% of the original osteotomy region (180 slices). A representative H and E stained histology image of the central region of the fracture is also shown. Scale bar in *lower left*-hand corner presents 500 μm in all histology images. 3D, three-dimensional. Color images are available online.

When comparing between all groups, there were significant differences in BV (*p* = 0.033) (Fig. 1), Tb.Th (*p* = 0.003) (Fig. 2), TotPor (*p* = 0.043), and BV/TV (*p* = 0.043). All treated groups had greater bone formation than control, other than GCSF-AMD, which had a negative impact on healing. However, only PBS-AMD reached statistical significance for increased overall BV and IGF1-AMD for percentage bone (BV/TV) within the callus. Notably, all groups had significant increases in Tb.Th other than VEGF-AMD. 3D reconstructed images of the representative groups are shown in Figure 3. For full microCT quantitative morphology results, see Table 2.

### Histomorphometric analysis

**FIG. 4.**
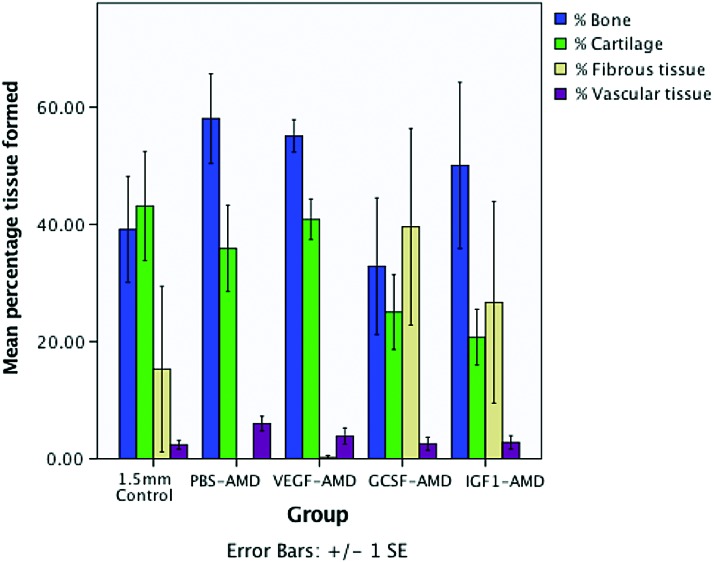
Mean ± SEM percentage tissue formed within the osteotomy from 2.5 × magnification histomorphometry. Color images are available online.

The 2.5 × histomorphologic analysis corroborated the microCT data; however, the differences were not statistically significant for percentage cartilage (*p* = 0.053) and percentage fibrous tissue (*p* = 0.059) for the PBS-AMD compared with controls. Patterns of increased bone formation were associated with decreased cartilage in groups with improved healing, whereas the worse performing groups had an increase in fibrous tissue with decreasing bone and cartilage formation (Fig. 4). Vascularization of the tissues was not significantly different, although animals in the GCSF-AMD group had the lowest levels of vascularization, whereas groups with more bone formation had higher levels of vascularization. However, the method of assessment was relatively nonspecific, and immunohistochemistry staining for CD31, α-SMA, or other endothelial markers would be a means to make a more comprehensive assessment of vascularization.

**FIG. 5.**
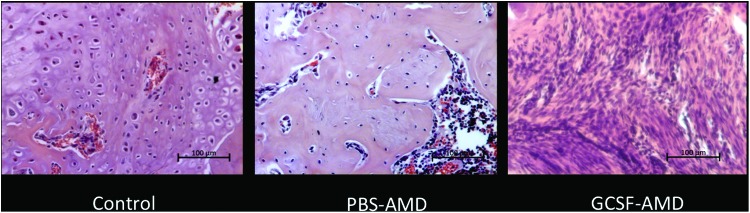
Histology of the central region of the fracture callus (hematoxylin and eosin), showing a large area of cartilage and hypertrophic chondrocytes adjacent to osteoid in the control; reduced cartilage, and increased woven bone formation in the PBS-AMD group, indicating increased endochondral ossification, and a highly cellular granulation tissue in the GCSF-AMD group. Scale bar in *lower right*-hand corner represents 100 μm in all images. Color images are available online.

Although not quantified, the cartilage tissue present in the GCSF-AMD group was observed to have fewer hypertrophic chondrocytes than the other groups (Fig. 5), suggesting reduced or slowed endochondral ossification. When comparing the controls to the best and worst performing groups, the control groups had a large area of cartilage in the central region, whereas the AMD3100 treated group had increased woven bone, but the GCSF group had a predominance of a highly cellular granulation type tissue (Fig. 5). The PBS-AMD group, which had the highest levels of bone and vascular tissue, had reduced cartilage and no fibrous tissue (Fig. 4). However, in other groups, cartilage formation was increased, suggesting conversion to bone by endochondral ossification (Fig. 4). The next highest bone formation was seen in VEGF-AMD, which also showed a low level of fibrous tissue and higher level of vascular tissue on histomorphometric analysis.

## Discussion

This study was the first to evaluate the potential effects of stem/progenitor mobilization in compromised fracture healing in rats, and demonstrated that AMD3100 antagonism in the early inflammatory phase of fracture healing has a beneficial influence on bone formation. Other studies have shown similar benefits in mice, but critically this study allowed for direct comparison of different pretreatment protocols, and for the first time demonstrated efficacy of endogenous mobilization in a mechanically standardized delayed union model. In this model of delayed union, there were significant increases in bone content within the fracture and a reduction in uncertain and not united radiographic categories. This confirms that this strategy can improve compromised fracture healing; however, because the animals were terminated after 5 weeks, it is unclear whether this strategy could avoid a nonunion forming. Nonetheless, there may be translational benefit for treating at risk groups of nonunion, such as tibial, humeral, or clavicular fractures.^27^

All strategies tested other than GCSF-AMD did improve fracture healing. AMD3100 without growth factor pretreatment gave significant increases in bone formation as measured on microCT, with a bigger (proportionally mineralized) callus compared with controls. This is similar to the findings of Toupadakis *et al.*,^24^ who gave AMD3100 only, but over three sequential days, rather than as a single dose. Although not performed here due to the high complexity, parabiotic studies with recapitulated labeled bone marrow^13,14^ would offer a mechanistic understanding of the exact processes leading to improved fracture healing.

Prior mobilization studies have shown in mice that pretreatment with VEGF or IGF1 preferentially increased the numbers of MSCs mobilized into the peripheral circulation.^20,23^ Based on these studies, we hypothesized that these growth factors would have a greater influence on fracture healing than giving AMD3100 alone, which comparatively mobilizes lower levels of MSCs, EPCs, and hematopoietic stem cells. The results of this study suggest that AMD3100 is the most effective protocol to improve fracture healing, bringing into question whether total numbers of different cells types, or their relative combinations, are more important. This study shows a conclusive benefit of AMD3100, which has been comprehensively shown to exert its main interaction at the CXCR4 receptor,^28,29^ which would lead to the release of cells that could enhance repair. However, it is impossible to totally exclude other mechanisms that may improve healing not due to stem/progenitor mobilization.

An issue with measuring circulating stem/progenitor cells is that they provide only a “snapshot” of the circulating pool of cells at the predicted peak elution time of 1 h postadministration of AMD3100.^28^ The duration and character of the profile of cell elution into the circulatory system is probably more important and so the true kinetics of the mobilization is unknown, and hence, although VEGF or IGF1 pretreatment before AMD3100 mobilized more MSCs at 1 h postadministration, this does not mean the total number is greater. It is also clear that mobilized cells home back to the bone marrow, or indeed other tissues such as liver, spleen, or lungs and, therefore, mobilization has to be considered as a highly dynamic and complicated process.^30^ Therefore, the only accurate evaluation of the potential of endogenous mobilization for fracture healing is with an *in vivo* fracture model.

Three mouse studies have also shown AMD3100 alone to improve bone formation,^21,22,24^ and this study in a rat model of delayed union further identifies AMD3100 along as more effective than combining with growth factor pretreatment. This may be due to physiological elevation of growth factors after fracture, including VEGF and IGF1,^31^ or that the mobilization profile, including cell types, timing of mobilization, and total numbers of cells, is most beneficial with AMD3100 alone. Toupadakis *et al.* gave three sequential doses of AMD3100 after fracture^24^ and studies on the elution and pharmacokinetics of AMD3100 show that serial administration will induce a peak mobilization at 1 h post-treatment to the same level each time, suggesting that receptor/system desensitization does not occur.^28^ This may mean that the number of stem/progenitor cells available to home to the fracture site could be further increased, but this remains to be demonstrated as beneficial over a single dose.

Another consideration that has not yet been addressed is the time of delivery of AMD3100, and presumably this has to be associated with SDF1 release at the fracture site and the maturity of tissue in the fracture gap. Consideration of the effect of CXCR4 blockade at the recipient fracture site also needs thought, as protracted treatment with AMD3100 throughout fracture healing or during distraction osteogenesis reduces healing.^12,32^ AMD3100's short half-life of 0.9 h in rodents^18^ likely underlies the benefit of short-lived blockade early in fracture healing, as it does not persist and inhibit ongoing migration of cells into the fracture site. AMD3100 therapy deserves further evaluation as the route to clinical translation is relatively simple, being already licensed for hematopoietic stem cell mobilization.

IGF1 pretreated groups showed the development of a relatively more mineralized callus with a significant increase in percentage bone. Kumar and Ponnazhagan^23^ evaluated IGF1 with AMD3100 in a mouse model and showed a significant increase in fracture bone mineral density on DXA scan, similar to the BV/TV in this study. They also showed that IGF1 alone gave a moderate improvement in bone density, whereas AMD3100 alone did not.^23^ This is in contrast with this study and the differences may relate to peculiarities of their model and species. As the only other group with a significant increase in bone formation, IGF1 and AMD3100 combined may also warrant further investigation.

As VEGF preceding AMD3100 has previously been demonstrated in mouse models to release the largest number of MSCs, it was hypothesized that maximal mobilization of these cells would be facilitated by the administration of VEGF and this would lead to the greatest bone healing. However, pretreatment with this growth factor did not show a significant increase in healing unlike AMD3100 alone or pretreatment with IGF1. This would suggest that the differential mobilization from this combination was less beneficial than the mobilization profile from AMD3100 alone. As hypoxia and subsequent vascularization of tissues within the fracture site plays a crucial role in progressive fracture healing and VEGF is a potent angiogenesis promoter with a role in endochondral and intramembranous bone formation,^33,34^ there was an expectation that VEGF would have beneficial effects.

Indeed, local delivery of VEGF in rabbit mandibular defects showed increased density of bone formation, although not the quantity.^35^ Histomorphometric assessment of vascularization was not the objective in this study, but notably the AMD3100 group had the highest number of blood vessels, although significant differences were not detected between groups. The VEGF pretreated group had the second highest percentage vascularized tissue, but the significance of that is difficult to know. In any case, there appears no significant advantage over AMD3100 for improving bone formation.

Pretreatment with GCSF before AMD3100 reduced fracture healing, which has not been previously shown. Interestingly, this group had a significant increase in percentage bone, which was indicative of a much smaller overall callus that proportionally had a higher BV component compared with controls. This bone region also had increased Tb.Th and reduced porosity. All treatment groups, including GCSF-AMD, had increased Tb.Th indicating thicker woven bone formation, but in the GCSF-AMD group the smaller callus had bone present that was structurally more dense. The reduced BV may relate to the less mature chondrocytes seen histologically, indicative of delayed endochondral ossification, which may in turn be due to excessive inflammation from mobilized inflammatory cells.

Increased presence of hematopoietic lineage osteoclast precursors, leading to bone reduction rather than deposition is also possible. Histologically, GCSF-AMD had the lowest level of bone, cartilage, and vascular tissue, and the highest level of fibrous tissue, suggesting a pattern of reduced endochondral ossification, reduced blood supply, and fibrous tissue development. Pitchford *et al.*^20^ showed that GCSF-AMD induced mild mobilization of MSCs and EPCs, but was principally a very effective mobilizer of hematopoietic stem cells and neutrophils. It is possible that the increased influx of neutrophils may have affected the progression of inflammation at the fracture site, preventing healing. CD34+ cells, which are a particularly well-represented population when mobilization is performed with GSCF ± AMD3100, are considered a population enriched in EPCs and hematopoietic stem cells. Transplantation of these cells has shown improved healing in several studies.^36,37^ However, this selected CD34+ population are a subset, which may explain the differences in healing seen, compared with mixed mobilized populations that include CD34+ cells. This has been borne out by studies showing a mixed GCSF mobilized mononuclear cell fraction being less efficacious than a subselected CD34+ population^38^ and excessive inflammation associated with the mononuclear cell population was suggested to be the cause. One study, however, has shown improved fracture healing with GCSF treatment alone, given on five consecutive days. Interestingly, their study lasted 200 days and significant differences were not seen until at least 20–30 days, with a reduction in the osteotomy gap distance. BV was significantly increased from ∼30 days, but all rats went on to nonunion.^39^

In conclusion, AMD3100 significantly increased fracture healing in a delayed union femoral model and was superior to protocols with growth factor pretreatment. This would suggest that peak MSC mobilization protocols previously identified are not solely beneficial for fracture healing; however, further study is required. In contrast, pretreatment with GCSF, which preferentially mobilizes hematopoietic stem cells and neutrophils had a negative effect on fracture healing and should be avoided. Further evaluation of the timing, dose, and frequency of administration of AMD3100 is warranted as it potentially offers a rapid route to clinical translation.
